# Supplementary material for: Flow chamber staining modality for real-time inspection of dynamic phenotypes in multiple histological stains
Source: PLoS One. 2023 May 4;18(5):e0284444. doi: 10.1371/journal.pone.0284444 (PMC10159194; doi:10.1371/journal.pone.0284444)
Supplement: S1 File — (DOCX) [file pone.0284444.s001.docx]

**Supporting Information**

**Flow chamber staining modality for real-time inspection of dynamic phenotypes in multiple histological stains**

**1. Supplemental materials and methods**

**S1 Table. Bodyweight (g) of mice used for related or repeated experiments (6 rounds).**

| **Organs** | **First** | **Second** | **Third** | **Fourth** | **Fifth** | **Sixth** |
| --- | --- | --- | --- | --- | --- | --- |
| **Heart^1^** | **23.9#** | **23.3#** | **21.1§** | **21.7§** | **20.3§^3^** | **20.3§^4^** |
| **Esophagus^2^** | **23.5** | **21,0** | **22.9** | **21.7** | **22.9** | **23.0** |
| **Brain^1^** | **21.7§** | **20.3§** | **20.3§** | **21.1§** | **21.1§** | **21.1§** |

The animals were delivered from Charles River Laboratory (Sulzfeld, Germany). 1. The organs were derived from male C57 BL/6J mice at the age of # 10 weeks and § 8 weeks. 2. The organs were derived from 41-week-old female BALB/c mice weighing 21.0 - 23.5 grams. 3. The brain was isolated for the experiments of HE coupled with histological special stains. 4. The lung, liver, and kidney used in the current work were derived from.

**S2 Table. Procedures for traditional histological stains.** For frozen sections, thaw the sections for 30 min at room temperature and fix them in PFA for 10 min before the following steps were performed.

| HE staining | 1. Wash in deionized water. |
| --- | --- |
|  | 2. Immerse in Harris hematoxylin for 5 minutes. |
|  | 3. Wash in water. |
|  | 4. Blue in running tap water for 10 minutes. |
|  | 5. Immerse in eosin for 40 seconds. |
|  | 6. Dehydrate sections with two 20-second washes in 100% ethanol. |
|  | 7. Place in xylene for 30 seconds. |
|  | 8. Mount in Kanadabalsam. |
|  |  |
| PAS staining | 1.  Wash in deionized water. |
|  | 2.  Oxidize in 0.5% periodic acid solution for 8 minutes. |
|  | 3.  Rinse in distilled water. |
|  | 4.  Place in Schiff reagent for 8 minutes. |
|  | 5.  Wash in tap water for 5 minutes. |
|  | 7. Dehydrate sections with two 20-second washes in 100% ethanol. |
|  | 8. Place in xylene for 30 seconds. |
|  | 9. Mount in Kanadabalsam. |
| Sirius red staining | 1.  Wash in deionized water. |
|  | 3. Stain in Picro-Sirius red for 15 minutes. |
|  | 4. Wash in tap water for 2 minutes. |
|  | 5. Drain the water from the slides with tissues. |
|  | 5. Dehydrate in three changes of 100% ethanol. |
|  | 8. Place in xylene for 30 seconds. |
|  | 9. Mount in Kanadabalsam. |

Sections to be stained were indicated in Fig. 2. For the related stain materials, refer to Table 2.

[**S3 Table. Procedures of Immunofluorescence staining for Human IgG (Fc specific).**](#_Toc241304027)

| 1. Let slides air dry at room temperature for 30 min. |
| --- |
| 1. Mark the slides with a PAP pen (Vector, Cat# H-4000) circling the sections. |
| 1. Fix in 4% PFA containing 0.5% triton for 10 min. |
| 1. Wash in PBST three times. 2. Incubate with Revacept (30 µg/ml) for 40 min at room temperature. 3. Wash in PBS three times. |
| 1. Incubate with the blocking medium for 1 hour. (Keep wet) |
| 1. Wash in PBS two times. |
| 1. Incubate sections with [biotinylated mouse anti-human IgG](http://www.ihcworld.com/products/ihc-detection-system/SPlink-APlink.htm) for 30 min. |
| 1. Rinse in PBS three times. |
| 1. Incubate sections in Streptavidin-FITC in PBS for 30 minutes.  (Protect slides from light). |
| 1. Rinse in PBS two times. (Protect slides from light). |
| 1. Coverslip with 20% glycerol. (Protect slides from light). |

Sections to be stained were indicated in Fig. 2. For the related stain materials, refer to Table 2.

- Sections for tests: Revacept-incubated sections.
- Sections of negative controls (no previous incubation of Revacept).
- Sections of blank controls (Revacept incubation but without antibody application).

[**S4 Table. Procedures of Immunofluorescence staining for CD45, Hemoglobin, and CD31.**](#_Toc241304027)

| 1. Let slides air dry at room temperature for 30 min. |
| --- |
| 1. Mark the slides with a PAP pen (Vector, Cat# H-4000) circling the sections. |
| 1. Fix in 4% PFA containing 0.5% triton for 10 min. |
| 1. Wash in PBST three times. 2. Retrieve antigens in 80% ethanol (containing 1% HCl) for 30 min. (for CD31 detection, optimal for the others) 3. Wash in PBS three times. |
| 1. Incubate with the blocking medium for 1 hour. (Keep wet) |
| 1. Wash in PBS two times. |
| 1. Incubate sections with [antibody](http://www.ihcworld.com/products/ihc-detection-system/SPlink-APlink.htm) for 30 min. |
| 1. Rinse in PBS three times. |
| 1. Incubate sections in biotinylated mouse anti-Rat IgG antibody for 30 minutes.  (skipped for Hemoglobin detection). |
| 1. Rinse in PBS two times. (Protect slides from light). 2. Incubate sections in Streptavidin-FITC in PBS for 30 minutes.  (Protect slides from light). 3. Rinse in PBS two times. (Protect slides from light). |
| 1. Coverslip with 20% glycerol. (Protect slides from light). |

Sections to be stained were indicated in Fig. 2. For the related stain materials, refer to Table 2.

- Sections for tests
- Sections of blank controls (without antibody application).

**S5 Fig. Preparation of flow chamber holder and assemblage of flow chamber setup.**

**Dish lid**

**Hole**

Main parts of flow chamber

**Preparation of flow chamber holder**

1. Drill two holes on the edge of a dish lid.
2. Screw the lid into a microscope object platform through the holes.
3. Demarcate the window of observation on the lid.
4. Fix the flow chamber setup within the observable window with an adhesive.

**2. Supplemental results**

**S6 Fig. Images of heart tissue captured at the beginning and end of 2-hour-perfusion, and comparison on mean intensity. A.** Heart sections were subjected to 2-hour perfusion, using a plotted flow velocity of 1 ml/min, with different solutions (H2O, 50%, and 100% ethanol), and photographed at high magnification (objective lens x 20) under a flow chamber setup. **B.** The similarity was tested on mean intensity measured at the initial and the end of the 2-hour-perfusion. The statistical analysis resulted in a statistically significant correlation (N = 18 pairs, Pearson’s r = 0.9836, P < 0.0001). Bars indicate 20 µm.

**S7 Fig. Images of heart tissue with hematoxylin stain.** The representative photos (three rounds) of hematoxylin-stained blue nuclei when contacted with slightly basic tap water and hematoxylin-stained red nuclei when contacted with acid ethanol solution were computationally registered in different regions of the heart. The corresponding images were blended with the blue-nucleus images overlapping the red-nucleus images. The red and blue colors of Hematoxylin-stained nuclei displayed at different pH values were well corresponded. The high magnification in localness is seen in No. 3. Bars indicate 10µm.

**S8 Fig. The images generated by IF and HE of FCS, the blending image, and the images by traditional staining procedures in lung tissue were demonstrated for comparison.** Targeted regions for inspection indicated by a blue inset in A2-C2 were magnified in A1-C1.

**S9 Fig. The images generated by IF and HE of FCS, the blending image, and the images by traditional staining procedures in liver tissue were demonstrated for comparison.** Targeted regions for inspection indicated by a blue inset in A2-C2 were magnified in A1-C1.

**S10 Fig. The images generated by IF and HE of FCS, the blending image, and the images by traditional staining procedures in kidney tissue were demonstrated for comparison.** Targeted regions for inspection indicated by a blue inset in A2 & B2 were magnified in A1 & B1.

**S11 Fig. The images generated by HE and special stains of FCS and traditional staining procedures in lung (A), liver (B), and kidney (C) tissues were demonstrated for comparison.** Targeted regions for inspection were indicated by a black inset.

**S12 Fig. A single section of the mouse brain was stained with HE, PAS, and Sirius red by FCS, and the outcomes were observed in Videos. A.** Flow chamber staining steps and time points for video acquisition. **B.** Targeted region for inspection, indicated by the black inset on a brain cross-section at Bregma 0 mm.

**S13 Fig. The images generated by HE and IF of FCS, the blending image, and the images of traditional staining procedures in heart tissue were demonstrated for comparison.** The targeted regions for inspection were indicated by blue insets in A2-C2 and magnified in A1-C1. The local magnification in A3-C3 was displayed in A2-C2.

**S14 Fig. The images generated by HE and IF of FCS, the blending image, and the images of traditional staining procedures in brain tissue were demonstrated for comparison.** The targeted regions for inspection were indicated by blue insets in A2 and magnified in A1. The local magnification in A3 was displayed in A2.
