# Supplementary material for: Flow chamber staining modality for real-time inspection of dynamic phenotypes in multiple histological stains
Source: PLoS One. 2023 May 4;18(5):e0284444. doi: 10.1371/journal.pone.0284444 (PMC10159194; doi:10.1371/journal.pone.0284444)
Supplement: Attachment — Submitted filename: Manuscript Number PONE-D-22-32143 .docx [file pone.0284444.s009.docx]

**Manuscript Number:** PONE-D-22-32143

**Article Type:** Research Article

**Full Title:** Flow chamber staining modality for real-time inspection of dynamic phenotypes in multiple histological stains

In this study, the authors introduced a new staining modality, flow chamber stain, which follows the current staining workflow but has additional features not found in conventional stains, allowing for (1) quick switching between destain and restain for multiplex staining in one section from routine histological preparation, (2) real-time inspecting and digitally capturing each stained phenotype, and (3) efficient staining. Hematoxylin-eosin (HE), Periodic acid–Schiff, Sirius red, and immunofluorescence for Human IgG, mouse CD45, hemoglobin, and CD31 were compared to its stains on microscopic pictures of mouse tissue (lung, heart, liver, kidney, esophagus, and brain). No substantial differences were found. The approach is accurate and reproducible in targeted stained section tests. Using the approach, immunofluorescence targets were easily located and visible structurally in HE- or special stained sections, and histological special or immune staining may be used to identify unknown or suspected components or structures.

However, the reviewer has some concerns:

Minor comments:

1) Figure 1 needs to be rectified. Not clear representation.
2) Abstract consists of few grammatical errors. Need thorough revision.
3) Page 4, “But the obvious…………..dropping”. Please reframe the sentence using appropriate words.
4) Page 4, line 104, replace the word ‘cover-slips’ with ‘coverslips’
5) Page 6, line 175-178, “Images of ………………..red-nucleus images. The sentence is not clear. Please use appropriate words with meaningful sentences.
6) Page 10, line 315, did you mean Tukey test? If yes? Please correct the word.
7) Page 12, line 362-363 please recheck the sentence and use appropriate words.
8) Page 19, table 2. Main reagents used for FCS and traditional staining, and Page 21, line 615. Please replace the word ‘Harri’s hematoxylin’ with ‘Harris hematoxylin’
9) In supporting Information- Table S3. Procedures of Immune Fluorescence stain for Human IgG (Fc specific). Did you mean ‘Immunofluorescence staining’, If yes, please correct the sentence everywhere accordingly.
10) Please maintain the same format (i.e.- Font size, theme and style) throughout the manuscript.

Major comments:

1. Please refer to the new existing similar technologies and define how the present work is different (pros and cons) from the existing technologies.
2. The work seems similar to the existing technologies e.g., Co-detection by indexing (CODEX). The author requires to justify the novelty of the present work with the similar existing technologies emphasizing the novelty of current work.
3. How did the author determine the flow rate to be 1 ml/min, and did they compare this value to other flow rates? If yes, please provide a brief description of the observation in the results section.
