# Supplementary material for: Flow chamber staining modality for real-time inspection of dynamic phenotypes in multiple histological stains
Source: PLoS One. 2023 May 4;18(5):e0284444. doi: 10.1371/journal.pone.0284444 (PMC10159194; doi:10.1371/journal.pone.0284444)
Supplement: Attachment — Submitted filename: Response to Reviewers.docx [file pone.0284444.s011.docx]

Response to Reviewers

Reviewer #1
**COMMENT 1:**

I would like to thank the authors for their detailed responses, mostly they addressed all the comments, but I still have some minor comments:

**RESPONSE:**

*We would also like to express our sincere gratitude to the anonymous reviewer for his/her kind patience in helping us to improve the quality of the manuscript. With the insightful comments, the manuscript has significantly been made better. Thanks again.*

**COMMENT 2:**

Major concerns: 1- For my 1st query, I think the authors should add the information mentioned in response to reviewers to the manuscript but in brief, especially if there is any variables or factors that would affect the reproducibility or the quality of the results.

**RESPONSE:**

*That’s a good suggestion. We have added information on the factors that affect the staining outcomes to the revised manuscript. Please see Lines 385-393, Pages 12-13. The addition is marked with red.*

**COMMENT 3:**

Major concerns: 2- Again, I couldn’t find any videos in submitted revised manuscript.

**RESPONSE:**

*That is too bad. We have indeed uploaded the videos and checked the uploading list before approving the submission. Besides, the loading materials have been subjected to technical checks, ensuring everything including files, pictures, and videos is loaded completely and correctly, before handing over to the reviewer’s hands. If something was missing, the relevant staff of Plos one editorial would inform us.*

*We have mentioned what happened in our cover letter to the editor since we never meet such a case before and can do nothing about it. Maybe it is better for the reviewer her/his self to ask relevant persons of Plos One and they can help.*

*We are really sorry for that.*

**COMMENT 4:**

Major concerns: 3- In the results: the authors mentioned “Images generated by IF and HE of FCS and traditional staining procedures in the adjacent sections were demonstrated” Figs S4-S6, I noticed. 3- Although the signal localization is close in IF but there are a lot of differences in IF stained sections in both methods, please check CD31 in Fig. S4, this would be problematic in specific small target regions assuming that the staining was done on identical sections for validation. Please add your response to the discussion section specifically the 3rd query, may be additional to the limitation.

*We agree with the reviewer.
We have added the responses to Limitation section. Please see Lines 429, 430, 442-448, Page 14. The addition is marked with red.*

*Limitation portion had taken a rather large space within the Discussion section after adding if not separated, and therefore, we separated Limitation part from the Discussion section. To keep the manuscript coherent and complete, the isolated becomes a separate section – “5. Limitation” in red (Line 427, Page 14).*

**COMMENT 5:**

Major concerns: 4- The authors already mentioned a lot of limitation of FCS modality which can be limiting factor to adopt this technique. Did the authors test more than one antibody with this technique? Please indicate that the current work is limited to single-target detection combined with conventional histological stains under a common-used microscope in the manuscript.

*We agree on the point. We have included the description in the revised manuscript. Please see Lines 360-365, Page12. It is labeled red.*

Reviewer #2
**COMMENT 1:**

I am satisfied with the response of authors. The authors clearly demonstrated a novel technique which is a fusion method of traditional histological stains, including immune stains, and conventional flow chamber.

**RESPONSE:**

*Thank you, Dr. Sahi.*
